# The contribution of illness perception to psychological distress in heart failure patients

**DOI:** 10.1186/s40359-014-0050-3

**Published:** 2014-11-28

**Authors:** Karen Morgan, Amanda Villiers-Tuthill, Maja Barker, Hannah McGee

**Affiliations:** Department of Psychology, Division of Population Health Science, Royal College of Surgeons in Ireland, Dublin 2, Ireland; Perdana University, PU-RCSI School of Medicine, Block B & D Aras 1, MAEPS Building, MARDI Complex, Jalan MAEPS Perdana, 43400 Serdang Darul Ehsan, Malaysia

**Keywords:** Heart failure, Illness perceptions, Anxiety, Depression, Psychological distress

## Abstract

**Background:**

The influences on the psychological well-being of heart failure (HF) patients have received limited attention. Illness perceptions are a specific set of cognitive representations that have been shown to predict health-related outcomes in other patient groups. This study sought to explore the role of illness perceptions in the psychological well-being of HF patients by creating a profile of illness perceptions in HF and examining their relations with anxiety and depression.

**Methods:**

Participants were 95 consecutive outpatients. Indices of psychological well-being were depression and anxiety, measured using the Hospital Anxiety and Depression Scale (HADS). Illness perceptions were measured using the Illness Perception Questionnaire – Revised (IPQ-R). Functional status was also determined using the New York Heart Association (NYHA) classification.

**Results:**

Illness perceptions were associated with indices of psychological well-being. Regression analyses showed that illness perceptions accounted for a significant proportion of the variance in both depression and anxiety. The contribution of illness perceptions was greater than that made by traditional covariates (socio-demographic variables and functional status).

**Conclusions:**

Results highlight dynamic interrelations between perceptions of illness and mental health indices. They also suggest that in considering the role of illness perceptions in psychological well-being, the primary focus should be on the overall dynamic of an individual’s illness experience rather than on specific illness dimensions. Findings highlight the potential role of illness perceptions in depression and anxiety in HF. This has implications for interventions to maximise psychological well-being in this patient group.

## Background

### Heart failure and depression

Heart Failure (HF) is widespread in aging populations across the world (Riegel et al. 2009). In addition, prevalence rates of depressive disorders in various cardiological conditions are significantly higher than in healthy general population (Kapfhammer 2011). There are established links between depression and heart disease (Kent and Shapiro 2009) and a substantial body of research has highlighted the psychological problems of many patients with HF. The prevalence of depression and anxiety is high in chronic heart failure (10–60% depression; 11–45% anxiety); with rates of depressive disorders 2 to 4 times higher in chronic heart failure patients than in the general population (Ladwig et al. 2014) and higher in heart failure patients than other cardiac patient groups (Moser et al. 2010).

Heart failure is associated with reduced quality of life and psychological wellbeing (Steptoe et al. 2000; Ladwig et al. 2014) and comorbid depression is associated with worsening of HF symptoms, physical and social function, and quality of life (Rumsfeld et al. 2003), poor adherence to recommended dietary therapy (Luyster et al. 2009), rehospitalisation (Redwine et al. 2007), high healthcare costs (Riegel et al. 2009; Eastwood et al. 2012) reduced quality of life in patient spouses (Chung et al. 2009), and overall increased mortality (Sherwood et al. 2007). Patients with heart failure also suffer from an almost twofold increased risk of dementia (Ladwig et al. 2014). With such wide ranging effects the need to empirically investigate potentially modifiable factors in the aetiology of this depression is apparent.

### Risk factors for depression

Anxiety has been shown to predict health-related outcomes including physical function (Sullivan et al. 1997) and quality of life (Mayou et al. 2000) in other cardiac disease populations. However worse prognosis for HF patients is only predicted by depression (Jiang et al. 2004) yet anxiety and depression are highly correlated in HF patients, and so the factors leading to both anxiety and depression warrant further attention as potential treatment options.

Considering that symptoms of depression may go unnoticed in this patient group as they may be attributed to the HF itself (Ladwig et al. 2014), it is important that the known factors contributing to psychological wellbeing are considered.

These include a reported history of mental ill-health and New York Heart Association (NYHA) functional class, and co-morbid physical illness (diabetes and angina) (Haworth et al. 2005).

### Illness perception

When investigating the antecedents of depression, patients’ beliefs about their illness should be considered. The relationship between beliefs about illness and health-related outcomes has been widely studied and illness perceptions are accepted as generically important to patient behaviour and outcomes (Hagger and Orbell 2003). Many of these studies have been conducted from the perspective of a specific theoretical framework, namely, the self-regulation model (SRM) of health and illness (Leventhal et al. 1992). According to this model, patients’ construct their own representations or models which help them make sense of their experiences and provide a basis for their own coping responses. Beliefs about illness or illness perceptions are central to the SRM and incorporate five key components. These are beliefs about the nature (identity), time-course (time-line), personal impact (consequences), causal factors (cause), and feasibility of control or cure (control/cure) of the illness. These components have been extended by dividing control/cure into treatment control and personal control, and by dividing time-line into acute/chronic time-line and cyclical time-line. Furthermore, additional measures specific to emotional representations of the illness and illness coherence have also been included (Moss-Morris et al. 2002).

Addressing illness perception early on with patients provides the opportunity to improve the concordance between doctor and patient beliefs and can achieve significant positive influences on a wide range of outcomes from patient anxiety to perceived understanding of information provided (Broadbent et al. 2009). Illness perceptions are associated with depression and anxiety across a range of illness populations (Evers et al. 2001). Patient beliefs influence their satisfaction with the consultation and future healthcare use, and can affect subsequent self-management and quality of life (Petrie et al. 2007). Modifying patients’ illness perceptions has been shown to improve recovery following MI (Petrie et al. 2002) and other self-regulatory interventions in illnesses as diverse as diabetes and AIDS have also shown improved patient outcomes (Petrie et al. 2003).

### Psychological aspects of HF treatment

While some guidelines for the treatment of HF focus exclusively on the physical management (Swedberg et al. 2005) the German Heart Society’s Position Paper on the importance of psychosocial factors in cardiology provides evidence-based recommendations for the integration of psychosocial factors into cardiological care. They recommend that HF patients with depression or anxiety disorders should be offered psychoeducation as a part of primary psychosomatic health care, with further treatment options such as stress management training and psychotherapy considered as necessary (Ladwig et al. 2014). Given their potential for facilitating positive outcomes across a wide range of illness populations, the role of illness perceptions as modifiable risk factors for depression and anxiety in HF warrants research attention.

Psychological symptom severity has been shown to predict self-care in HF patients (Riegel et al. 2009) and recently a study by Goodman et al. reported that despite improved self-monitoring over time HF patients who reported a poor emotional state and a belief that their illness was outside their control failed to translate their self-monitoring into self-management, highlighting the importance of patients’ understanding and perceptions of their condition (Goodman et al. 2013). Work grouping patients by their illness perceptions suggests that reduced QoL and depression are more common in patients with certain types of illness perception (Le Grande et al. 2012). This study sought to make a novel contribution to the HF evidence-base by assessing HF illness perceptions and highlighting their importance for mental health.

To build on illness perception studies designed to demonstrate the associations of illness perceptions with behavioural and emotional outcomes of illness, effective interventions designed to change relevant dysfunctional illness perceptions and thus improve outcomes of illness are needed (Petrie et al. 2007).

### Aims and objectives

The specific aims and objectives of the study were as follows:

1. To create a profile of illness perceptions in the study population by examining the distribution of scores on illness perception subscales
2. To investigate the theoretical and practical significance of illness perceptions within the context of mental health by
  examining the relationship between illness perceptions and mental health indices, specifically depression and anxiety
  determining the contribution of illness perceptions to mental health indices after traditional covariates had been considered.

## Methods

### Design and participants

This study employed a cross-sectional design. A convenience sample of HF patients were recruited from cardiac outpatient clinics at three large urban hospitals in Ireland and Nothern Ireland. Patients were eligible for inclusion in the study if they had a primary diagnosis of HF as determined by the hospital medical team (for the purposes of this study an ejection fraction <40% was the criteria used to identify HF; diagnosis confirmed by echocardiogram); were not cognitively impaired (cognitive impairment was defined as scoring less than 8 on the Abbreviated Mental Test (AMT)); and were able to complete the study through English. Ethical approval was granted for this study by Queens University Belfast REC and the ethics committee Beaumont Hospital, the Adelaide and Meath Hospital REC.

### Procedure

One hundred and ten consecutive patients were approached; ninety five of these patients agreed to participate (response rate: 86%). Consent to approach patients was provided by their cardiologists at each hospital and contact details for eligible patients attending a routine outpatient appointment were forwarded by the medical team. Participants completed questionnaires via interviews conducted within two weeks of a hospital outpatient department appointment. Where feasible, following informed consent, interviews were carried out in the hospital. Some interviews were conducted in patients’ homes.

### Measures

*Socio-demographic indices* including gender, marital status, age, and level of education were recorded. Functional status was determined using the New York Heart Association (NYHA) classification (I-IV), as provided by the medical team. NYHA classes are sometimes referred to as mild (I), moderate (II), severe (III) and very severe (IV).

*Anxiety and depression were* assessed using the Hospital Anxiety and Depression Scale (HADS) (Zigmond and Snaith 1983). This 14-item scale comprises two subscales: anxiety and depression. Items are rated on a four-point scale, giving maximum scores of 21 for anxiety and depression. Scores of 11 or more on either subscale are considered to be a significant ‘case’ of psychological morbidity, while scores of 8–10 represent ‘borderline’ and 0–7 ‘normal’. The HADS is widely used, has good reliability and validity, and performs well in assessing severity and caseness of depression and anxiety in clinical and general populations (Bjelland et al. 2002).

The Revised Illness Perception Questionnaire (IPQ-R) (Moss-Morris et al. 2002) was used to assess *illness perceptions*. The subscales of the IPQ-R are timeline acute/chronic, timeline cyclical, consequences, personal control, treatment control, identity, illness coherence, emotional representations and cause. With the exception of the identity subscale, responses are rated on a 5-point scale. Scores on the identity subscale are the sum of symptoms attributed to HF. The IPQ-R has been used with a wide variety of patient groups. It has good reliability and validity and is successful in predicting different aspects of adaptation and recovery in chronic illness (Moss-Morris et al. 2002).

### Statistical analyses

Data was entered into SPSS 14.0.1 for Windows statistical package. All variables were found to approximate normal distribution. Internal reliability was assessed using Cronbach alpha (α); an alpha coefficient of .70 was used as the critical value for good internal reliability (Nunnally 1978). Pearson correlations were used to examine relationships between continuous variables and hierarchical regression analyses were conducted in order to determine the independent contribution of IPQ-R variables to psychological well-being.

## Results

The socio-demographic profile of study participants is outlined in Table 1.

**Table 1:** **Participant socio-demographic characteristics (n =95)**

**Socio-demographic characteristics**	
Age (years) (M ± SD)	73.1 (8.77)
Gender (%)	
Men	81.1
Marital status (%)	
Married	85.3
Separate/Divorced	3.2
Widowed	8.4
Never married	3.2
Education (%)	
Primary education only	18.7
Partial second level	52.7
Completed second level	20.9
Third level diploma/Primary degree/Higher degree	7.7

### Illness perceptions

Six of the nine IPQ-R subscales had good internal reliability. Subscale distributions can be seen in Table 2. Results suggest that there were no notable floor or ceiling effects for any IPQ-R dimensions.

**Table 2:** **Distribution of IPQ-R subscale scores (n =95)**

	**Number of items**	**Alpha**	**Range**	**Mean (SD)**	**Median**
Timeline acute/chronic	6	.71	1 - 5	3.78 (.70)	3.67
Timeline cyclical	4	.88	1 - 5	2.74 (.97)	2.50
Consequences	6	.61	1 - 5	3.40(.66)	3.50
Personal control	6	.69	1 - 5	3.41(.72)	3.5
Treatment control	5	.36	1 - 5	3.42 (.44)	3.4
Identity	14	.77	1 - 14	5.79(3.01)	5.0
Illness coherence	5	.83	1 - 5	3.52 (.80)	3.6
Emotional representations	6	.83	1 - 5	2.94 (.83)	2.6

Participants did not attribute a high proportion of symptoms to their illness (Identity mean score =5.79). They generally experienced their condition as being chronic (timeline acute/chronic mean score =3.77), having little variation (timeline cyclical mean score =2.74) and having a negative impact on their lives (consequences mean score =3.40). Participants also felt that their illness was controllable both personally (personal control mean score =3.41) and through treatment (treatment control mean score =3.42), they had a moderately negative emotional response to their illness (emotional representations mean score =2.94) and generally reported understanding their illness (illness coherence mean score =3.52). The illness perception sub-scales with the greatest number of patients recording scores at the upper end of the range (top 10% of possible scores) were timeline acute chronic (15.8%) and illness coherence (9.7%). The ‘treatment control’ (1.1%) and ‘identity’ sub-scales had fewest subjects with high scores. The remainder of sub-scales had 5.3-5.6% of patients scoring at the upper end of the range.

### Functional status

Functional status scores spanned the range (I-IV), with 32.6% in class I, 34.7% in class II, 26.3% in class IV and 6.3% in class V.

### Anxiety and depression

HADS subscales were internally reliable (α depression; .78; α anxiety; .89). Mean scores for depression and anxiety were 5.53 (SD 3.9) and 5.92 (SD 5.1). Almost one third (29.7%) of participants scored above the cut-off point of seven on the depression scale (13.2% possible cases and 16.5% probable cases) while close to a third (30.1%) scored above the cut-off point of seven on the anxiety scale (12.4% possible cases and 17.7% probable cases).

### Illness perceptions and depression

Preliminary bivariate relations between individual variables and depression scores are presented in Table 3. Results showed that lower levels of perceived control were associated with higher depression scores. Multiple linear regression models were also developed with depression as an outcome variable. Socio-demographics were entered on the first step (Model I), functional status was entered on the second step (Model II), and illness perceptions were entered on the third step (Model III). This sequence was adopted in order to determine the contribution of illness perceptions after accounting for traditional explanatory measures.

**Table 3:** **Zero-order correlations between demographic characteristics, illness perceptions and depression (HADS-D) and anxiety (HADS-A) (n =95)**

**Personal characteristic**	**Depression (HADS-D)**	**Anxiety (HADS-A)**
*Socio-demographic variables:*		
Age	-.096	-.126
Gender	.092	.007
Education	-.202	-.314**
*Functional status:*		
NYHA class	.158	.243*
*Illness perceptions:*		
Timeline acute/chronic	.077	.064
Timeline cyclical	.079	.210
Consequences	.034	.149
Personal control	-.371**	-.305**
Treatment control	-.180	.019
Identity	.214	.225
Illness coherence	-.043	-.083
Emotional representations	.200	.326**

**p < .01, *p < .05.

Table 4 displays the standardised regression coefficients (β), R, R^2^ and change in R^2^ after entering all variables. Model I (socio-demographic variables) explained 6.8% of the variance (adjusted R^2^) in depression scores. The overall effect of socio-demographic variables was not significant, (F (3, 60) =2.52, p = 0.066). Subsequent entry of Model II (functional status) did not explain any additional variance in depression, (F (4, 59) =1.907, p = .121). Interestingly NYHA class itself was not independently significant. Finally, when Model III (illness perceptions) was added a total of 35.3% of the variance (adjusted R^2^) (F (12, 51) =3.863 p < .05) in depression scores was accounted for.

**Table 4:** **Summary of hierarchical regression analysis explaining depression scores (n =84)**

**Parameter**	**Standardised regression coefficients**			**R**	**R** ^**2**^	**Change in R** ^**2**^
*(I) Socio-demographic variables:*	Model I	Model II	Model III	.335	.112	.112
Age	-.203	-.216	-.278			
Gender	.117	.122	.236			
Education	-.251	-.263	--.269			
*(II) Functional status:*				.338	.114	.003
NYHA class		.053	-.061			
*(III) Illness perceptions:*				.690	.476	.362**
Timeline acute/chronic			.184			
Timeline cyclical			-.028			
Consequences			-.142			
Personal control			-.176			
Treatment control			-.146			
Identity			.366			
Illness coherence			.064			
Emotional representations			.348			

**p < .01.

### Illness perceptions and anxiety

Preliminary bivariate analyses demonstrating the relationship between individual variables and anxiety scores are presented in Table 3. Results showed that higher anxiety scores were significantly associated with lower levels of perceived control over one’s condition, and with a more negative emotional response to one’s condition. The same sequence of modelling (Model I – socio-demographic, Model II – functional status and Model III – illness perceptions), was adopted as in the analysis of depression variables in order to determine the contribution of illness perceptions to anxiety after more traditional explanatory measures had been considered.

Table 5 shows the standardised regression coefficients (β), R, R^2^ and change in R^2^ after entering all variables. Model I R accounted for 5.8% of the variance (adjusted R^2^) in anxiety scores (F (3, 58) =2.261, p = .091). Model II, was significant, explaining an additional 8.5% of the variance (adjusted R^2^) in anxiety scores (F (4, 57) =3.536, p = .012). The addition of Model III (illness perceptions) explained a further 33.7% of the variance (adjusted R^2^) (F (12, 49) =5.699, p < .01) in anxiety.

**Table 5:** **Summary of hierarchical regression analysis explaining anxiety scores (n =84)**

**Parameter**	**Standardised regression coefficients**			**R**	**R** ^**2**^	**Change in R** ^**2**^
*(I) Socio-demographic variables:*	Model I	Model II	Model III	*.324*	*.105*	.105
Age	-.160	-.242	-.237			
Gender	-.008	.024	.043			
Education	-.290	-.359	-.368			
*(II) Functional status:*				*.446*	*.199*	.094*
NYHA class		.325	.092			
*(III) Illness perceptions:*				*.763*	*.583*	.384**
Timeline acute/chronic			.120			
Timeline cyclical			-.011			
Consequences			.025			
Personal control			-.213			
Treatment control			.062			
Identity			.042			
Illness coherence			-.128			
Emotional representations			.473			

**p<.01.

*p < .05.

## Discussion

Reported illness perception profiles in the study population indicated that participants typically experienced their HF as chronic, with little variation in HF symptoms, and had a good understanding of the condition. They also indicated a sense of personal control over their HF and tended to believe in effectiveness of treatment; this is encouraging given evidence that control-related cognitions can have protective effects on health-related outcomes. The cure/control dimension of illness perception refers to the sense of empowerment regarding effectiveness of coping behaviours (Hagger and Orbell 2003) and is likely to influence a patient’s motivation for self-care. In other areas of cardiac health it has been shown that regaining control was the core process in adjustment after an acute event (Cherrington et al. 2004).

It is concerning, however, that the perceived personal consequences of HF and the extent of negative emotional responses HF generated for participants were considerable. These negative illness perception responses are noteworthy as they have been shown to negatively influence emotional adjustment and health-related outcomes across other severe and chronic illness populations. The odds of experiencing a complication in post myocardial infarction patients increased with more negative representation of illness (Cherrington et al. 2004), and illness perceptions before rehabilitation predicted exercise capacity and psychological well-being in COPD patients (Zoeckler et al. 2014). Additionally, in cancer patients negative illness perceptions were associated with non-adherence to treatment (Iskandarsyah et al. 2014).

In relation to identity, participants on the whole did not attribute a wide range of ‘symptoms’ to their HF. This suggests that they have specific beliefs as to what physical experiences are HF-related. Similarly, they did not convey strong beliefs about HF causal factors, implying a specificity of beliefs about the origin of their condition. This is an important consideration in the treatment of HF as the development of a shared understanding of disease between patient and practitioner is beneficial in facilitating decision making, formulating therapeutic plans that are acceptable to the patient and hence improving treatment adherence (Street and Haidet 2011).

Exploration of the relationship between illness perceptions and depression and anxiety provides insight into the theoretical and practical relevance of illness perceptions in the context of mental health. Interrelations between illness perceptions and depressive symptoms were highlighted by the significant correlation seen between individual dimensions of the illness experience and depression scores. Specifically, assumptions of control were associated with lower depression and anxiety scores while negative emotional responses were associated with higher anxiety scores and a significant proportion of the variance in both anxiety and depression could be accounted for by illness perceptions, even after socio-demographic variables and functional status had been considered.

Furthermore, for both depression and anxiety, illness perceptions explained more variance than both socio-demographic variables and functional status together. This may suggest that that the beliefs individuals hold about their HF are potentially more important than traditional explanatory variables in accounting for anxiety and depression. However the challenge of measuring illness perceptions independently of depression and anxiety must be addressed. There is potential for the items included in the IPQ-R that measure emotional representations to be capturing the same information that is targeted by the depression items on the HADS. For example the IPQ-R items that refer to feelings of anxiety, depression and a lack of hope caused by the illness ask about feelings also described in HADS items. While this potential for the two measures to be effectively measuring aspects of the same thing potentially accounts for some of the correlation between the emotional representation subscale and the mental distress indices, the fact that these emotions are an integral aspect of illness perception highlights the role of illness perception assessment as a method of screening for those patients who may be sub-clinical on depressions and anxiety screening measures, yet remain at risk.

In the Self-Regulation Model (SRM) the separate concepts of illness perceptions and psychological distress are addressed independently while still acknowledging their mutual influence. This model explores the interrelationship between illness, illness perceptions, coping processes, and health outcomes. According to SRM anxiety and depression are outcomes –products of coping which depends on illness perceptions. Individual’s illness outcomes, including anxiety and depression, are determined by several factors. The first is their experience of illness, from which individuals generate their own perceptions or cognitive and emotional representation of the illness, which in turn influences the choice and engagement in various coping styles and eventually psychological distress, with each factor feeding back to the others (Leventhal et al. 1992). Unfortunately in a cross-sectional study such as ours the relationship cannot be teased out, so we recommend a longitudinal study to allow these pathways to be explored in detail.

Mediation of the relationship between illness perception and depression, anxiety and stress by maladaptive rumination has been described by Lu et al. (2014). They found that maladaptive rumination connected perceived identity, consequences of illness and emotional representation of illness and negative emotions with negative emotional outcomes, suggesting that although they are similar concepts encompassing many similar attributes there is an identifiable mechanism by which one can affect the other. The close interplay between illness perceptions and depression and anxiety has also been highlighted by Zyrianova et al., identifying illness perception as mediator in the relationship between depression, anxiety and pain (Zyrianova et al. 2011). In addition illness perceptions are formed directly in response to an illness and influence coping skills developed to deal with that illness, rendering them illness specific. The HADS on the other hand is not specifically focused on a particular illness – it gathers a snapshot of the previous 2 weeks encompassing a more holistic sphere of influence on mental distress indices.

In their study on the effect of depression and perceptions about heart failure on quality of life in patients with advanced heart failure Hallas et al. reported a similar confluence of more negative beliefs about the consequences of HF and less perceived control over symptoms leading to maladaptive coping styles such as denial and behavioural disengagement, with more severe levels of depression and anxiety (Hallas et al. 2011). While the challenge of independently assessing illness perceptions and depression remains, they suggest that focusing on changing negative beliefs may improve the psychological well-being and quality of life of HF patients. This suggestion and others that enhancing control cognitions and positive focus coping may be important in improving psychological health (Dempster et al. 2011) advocate the merit of illness perception as a treatment goal in its own right.

Other socio-demographic risk factors cited in the literature for depression among HF patients include low educational attainment, being unmarried or living alone, and being unemployed or having a low income, and unhealthy behaviours such as alcohol and tobacco use, poor nutrition, sedentary lifestyle and poor adherence to medical regimens are also associated with depression in HF patients (Eastwood et al. 2012). In consensus with the findings of this study a recent study by Eastwood et al. showed increased anxiety and low perceived control as predictors of depression in both men and women with HF (Eastwood et al. 2012).

These findings align with empirical evidence highlighting the significance of illness perceptions in depression and health related quality of life across a range of illness populations (Cherrington et al. 2004; Hyphantis et al. 2013; Skinner et al. 2014; Hudson et al. 2014; Spain et al. 2007; Edgar and Skinner 2003), and provide support for the effect of cognitive and emotional factors on depression in HF (Klein et al. 2007; Jiang et al. 2004). Illness perception has also been acknowledged as a notable factor in adjustment to chronic disease (de Ridder et al. 2008).

Although anxiety has been described as a predictor of depression, to date, no studies have specifically investigated the role of psychological factors in anxiety in HF. As such, the present findings make an important and novel contribution to the HF evidence-base. The role that illness perceptions may play in anxiety in HF is consistent with studies documenting the role of illness perceptions in anxiety across a range of other illness populations (Edgar and Skinner 2003; Jopson and Moss-Morris 2003).

These findings support the theoretical relationship between a pattern of illness beliefs and mental health outcomes. In keeping with the overall premise of the SRM which proposes a holistic view of the illness experience, the finding that no one dimension of illness perception was independently significant in explaining both depression and anxiety suggests that the significance of individual dimensions should not be over-emphasised. According to the SRM, the role that illness beliefs may play in health-related outcomes is focused primarily on the overall dynamic of an individual’s illness representation, i.e. system of illness beliefs, rather than on specific dimensions. At a practical level, these findings suggest that illness perceptions can potentially be included in the list of psychological risk factors for depression and anxiety in HF patients.

The finding that perceived control is an important correlate of depressive and anxiety symptoms has implications for the development of patient centred interventions aimed at increasing patient empowerment. Interventions focusing on patient empowerment and enablement may improve perceptions of personal controllability of disease and positively influence outcomes by engaging the patient in their own care. In their critique of patient empowerment Aujoulat et al. argue that empowerment can be achieved through either ‘holding on to’ self-representations and the associated control of the disease or the converse ‘letting go’ and acceptance that not everything is controllable (Aujoulat et al. 2008).

There are few interventions developed to change illness perceptions, one notable example is the trial of a psychological family-based intervention to change illness perceptions in patients with poorly controlled type 2 diabetes, in which a the intervention group showed a significant changes in illness perceptions and improvement in HbA1c (Keogh et al. 2011). However there have been no such interventions to date in HF; this is an area of interest for future research.

Cognitive-behavioural therapy (CBT) for depression and/or anxiety in HF patients might beneficially shape illness perceptions towards a more positive emotional response. Specifically, in addition to addressing negative illness cognitions CBT might also attempt to foster more positive illness cognitions such as perceived personal control over illness. This approach would be in keeping with proposals to expand and develop CBT to incorporate positive psychological principles (Riskind 2006). The use of illness perception assessment in HF patients may assist in identifying those patients who would benefit from CBT despite not falling into a probable case of depression or anxiety. Alternatively short psychological interventions aimed specifically at changing illness perceptions such as that used by Keogh (Keogh et al. 2011) would be an alternative option to CBT for depression and anxiety in HF patients with unfavourable illness perception profiles.

Petrie et al. showed that an intervention aimed at changing illness perceptions in post myocardical infarction (MI) patients achieved significant positive changes in patients’ views and functional outcome (Petrie et al. 2002). Similar treatment interventions aimed at enhancing and/or modifying specific illness perceptions such as personal control in HF patients may be a worthwhile goal in preventing and treating depression and anxiety in this group.

Several potential limitations of the study deserve attention. Firstly, the cross-sectional nature of this study limits the extent to which conclusions may be drawn. For instance, the causal relationships between illness perceptions and psychological well-being cannot be determined. Although prospective and experimental studies with other illness populations are consistent with the assumption that illness perceptions influence adaptive outcomes (Hagger and Orbell 2003), it is likely that multiple and reciprocal causality is involved. As such, further work is needed to investigate changes in illness perceptions over time and to determine the temporal relationship between illness perceptions and anxiety and depression in the context of HF.

The reliance on self-report methods in the assessment of psychological well-being may also be a limiting factor in the present research. Despite their subjectivity, however, self-report measures can predict morbidity and mortality (Bruce and Fries 2003) and as such are a practical compromise to more rigorous but time-consuming multi-modal assessments.

The sample population in this study was a consecutive sample and was predominantly male (81.1%). This is not representative of national HF rates which although higher in men are more evenly distributed between the sexes than in this sample population (Ho et al. 1993) and so influences the generalizability of the results.

The poor internal reliability of three subscales of the IPQ-R including ‘treatment control’, ‘personal control’ and ‘consequences’ must be counted as a limitation, especially in light of the potential we have suggested for the addressing of ‘personal control’ perceptions through interventions. Finally, in keeping with recommendations (Cohen 1990), the complexity of regression analyses was minimised by restricting the number of explanatory variables. Although Cohen argues that this is likely to yield more meaningful and comprehensible results, it should be noted that there are other variables that might have accounted for a greater proportion of the variance in depression and anxiety.

## Conclusion

In conclusion, illness perceptions hold a principal role in elucidating depressive symptomatology and anxiety in HF, relative to other known covariates. As such, patients’ illness perceptions should be addressed as a primary modifiable component in the development of depressive disorders in HF. High prevalence of depression in HF and its associated increase in morbidity and mortality warrants the continued development of a relevant and critical evidence-base with regard to the role of illness perceptions in the mental well- being of HF patients. This in turn will promote the development of novel approaches to the management and prevention of anxiety and depression in HF and in doing so, foster significant reforms in mental health care delivery.
